# Prognostic value of myocardial perfusion imaging in asymptomatic high-risk diabetic patients: 10-year follow-up of the prospective multicentre BARDOT trial

**DOI:** 10.1093/ehjci/jeaf126

**Published:** 2025-04-23

**Authors:** Kathrin Thommen, Simon M Frey, Daniel Kammerer, Damian Wild, Philip Haaf, Felix Mahfoud, Michael J Zellweger, the BARDOT investigators, Jan Müller-Brand, Miriam Brinkert, Thilo Burkard, Federico Caobelli, Stefanie von Felten, Gianluca Haenny, Raban Jeger, Ulrich Keller, Michael Mauraun, Hans H Osterhues, Otmar Pfister, Matthias Pfisterer, Naina Rastalksy

**Affiliations:** Department of Cardiology, University Hospital Basel, University of Basel, Petersgraben 4, Basel CH-4031, Switzerland; Department of Cardiology, University Hospital Basel, University of Basel, Petersgraben 4, Basel CH-4031, Switzerland; Cardiovascular Research Institute Basel (CRIB), University Hospital Basel, University of Basel, Spitalstrasse 2, Basel CH-4056, Switzerland; Department of Internal Medicine, District Hospital of Schopfheim, Schopfheim, Germany; Division of Nuclear Medicine, University Hospital Basel, University of Basel, Petersgraben 4, Basel CH-4031, Switzerland; Department of Cardiology, University Hospital Basel, University of Basel, Petersgraben 4, Basel CH-4031, Switzerland; Cardiovascular Research Institute Basel (CRIB), University Hospital Basel, University of Basel, Spitalstrasse 2, Basel CH-4056, Switzerland; Department of Cardiology, University Hospital Basel, University of Basel, Petersgraben 4, Basel CH-4031, Switzerland; Cardiovascular Research Institute Basel (CRIB), University Hospital Basel, University of Basel, Spitalstrasse 2, Basel CH-4056, Switzerland; Department of Cardiology, University Hospital Basel, University of Basel, Petersgraben 4, Basel CH-4031, Switzerland; Cardiovascular Research Institute Basel (CRIB), University Hospital Basel, University of Basel, Spitalstrasse 2, Basel CH-4056, Switzerland

**Keywords:** coronary artery disease, asymptomatic type 2 diabetes mellitus, myocardial perfusion, silent myocardial ischaemia, screening, revascularization

## Abstract

**Aims:**

Patients with type 2 diabetes mellitus (T2DM) are at high risk for coronary artery disease (CAD) related events and are often asymptomatic. The role of cardiac imaging in screening asymptomatic T2DM patients remains controversial, as existing studies are limited by short follow-up periods. Therefore, study aim was to provide long-term (10 years) outcome data of T2DM patients screened with cardiac imaging.

**Methods and results:**

A total of 400 asymptomatic high-risk T2DM patients without history of CAD underwent screening with Single Photon Emission Computed Tomography (SPECT). Abnormal SPECT was defined as Summed Stress Score ≥ 4 or Summed Difference Score ≥ 2. Patients were followed for all-cause mortality and major adverse cardiovascular events (MACE, cardiovascular mortality + myocardial infarction).

The mean age was 63 ± 8 years; 69% were male. Diabetic end-organ damage was present in 87% of patients. Baseline SPECT was abnormal in 22% of patients. Median follow-up was 11.1 (8.8, 12.8) years. Abnormal SPECT was associated with higher all-cause mortality [hazard ratio (HR) 1.614, *P* = 0.029] and MACE (HR 2.024, *P* = 0.009). A normal SPECT was associated with a significantly better prognosis (all-cause mortality 1.9 vs. 3.1%/year, *P* = 0.016; MACE 1.2 vs. 2.3%/year, *P* = 0.010). In the small subgroup of patients with abnormal SPECT, the treatment strategy (revascularization vs. conservative) had no effect on event-free survival.

**Conclusion:**

A normal SPECT was associated with an excellent long-term prognosis in high-risk T2DM patients. Hence, SPECT could serve as a valuable tool for advanced risk stratification in this population.

## Graphical Abstract

**Graphical Abstract:**
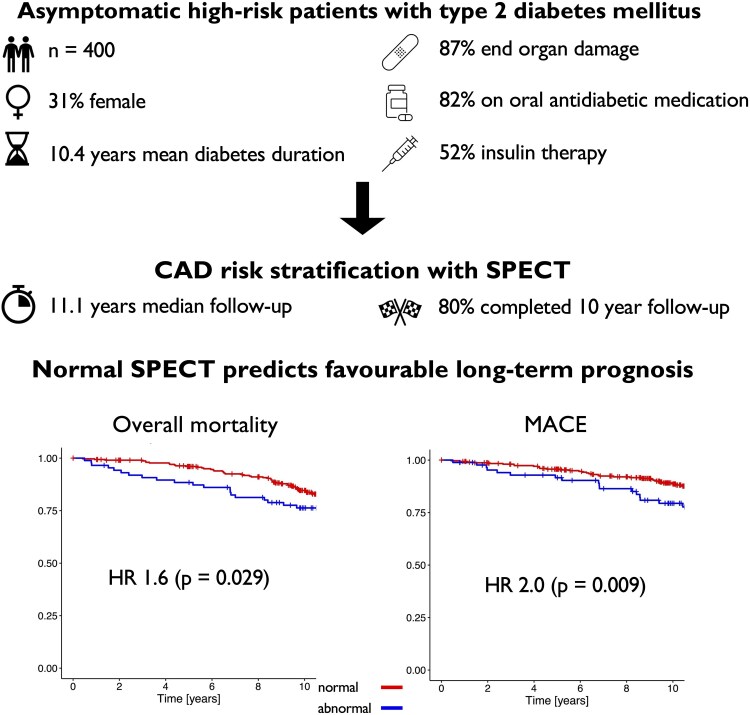

## Introduction

Type 2 diabetes mellitus (T2DM) is a major cardiovascular risk factor, with its global prevalence projected to exceed >7% by 2030^1^. Several studies have demonstrated that both diabetic and pre-diabetic patients face an increased risk of coronary atherosclerosis and major adverse cardiovascular events (MACE).^2,3^ Additionally, abnormal myocardial perfusion and ischaemia have been found more frequently in asymptomatic compared with symptomatic diabetic patients.^4^ Hence, individuals with diabetes are considered at high risk for coronary artery disease (CAD). However, with the expansion of screening programs and the availability of effective treatment options, traditional risk stratification approaches have come under scrutiny.^5^ Consequently, considerable efforts have been made to identify patients at high-risk for CAD and enhance risk assessment using imaging modalities, including Single Photon Emission Computed Tomography (SPECT).^6–8^

Current European Society of Cardiology guidelines suggest that coronary computed tomography (CT) or functional imaging may be considered in asymptomatic patients with diabetes mellitus for screening of CAD (Class IIb Evidence Level B).^9^ In contrast, the American Diabetes Association advises against screening for CAD in diabetic patients, arguing that screening does not improve outcomes if cardiovascular risk factors are already being treated.^10^ This recommendation may have been influenced by a meta-analysis that found no significant reduction in cardiac mortality through non-invasive CAD screening.^11^ However, the included studies had a relatively short observation period (3.6–4.8 years). It is possible that longer follow-up could have revealed different outcomes. Hence, long-term outcome data from non-invasively screened T2DM patients are crucial to better inform imaging-based screening strategies in such patients.

Herein, we report the 10-year follow-up results from the prospective, multi-centric, randomized Basel Asymptomatic high-Risk Diabetics’ Outcome Trial (BARDOT), which assessed the diagnostic and prognostic value of an imaging-based screening program using SPECT in asymptomatic high-risk T2DM patients.

## Methods

### Patients

Four hundred patients with T2DM but without known CAD or symptoms suggestive of CAD, were enrolled, as previously reported.^8,12^ Briefly, enrolment criteria included high coronary risk defined as end-organ damage (such as peripheral or carotid occlusive disease, retinopathy, microalbuminuria, or autonomic cardiac neuropathy as measured by Ewing *et al.*^13^) or a combination of age over 55 years, diabetes duration of at least 5 years, and at least two additional cardiac risk factors (positive family history of CAD, smoking, hypercholesterolaemia, and hypertension). Exclusion criteria included NYHA Class IV, life expectancy below 3 years and age above 75 years. From June 2004 to December 2010, a total of 2063 outpatients were screened at the University Hospital Basel, Switzerland, the District Hospitals Lörrach and Schopfheim in Germany and by office-based diabetologists. Of these, 1136 (55%) did not meet the enrolment criteria and 527 (26%) declined participation, resulting in a final cohort of 400 participants (19%). The trial was approved by the local Ethics committees of all participating centres and all patients gave written informed consent (Ethik-Kommission beider Basel, project ID 88/02; Ethik-Kommission Landesärztekammer Baden-Württemberg project ID 2006-001430-40).

### Study design

As previously published,^8,12^ the BARDOT protocol included a clinical evaluation and rest/stress SPECT at enrolment and after 2 years. Follow-up assessments were conducted through phone interviews, letters, and medical records after 5 and 10 years. Participants with abnormal SPECT at baseline were randomized to either pharmacological treatment only (conservative arm) or revascularization plus pharmacological treatment (invasive arm).

### Single photon emission computed tomography

All participants underwent SPECT imaging at the Division of Nuclear Medicine at the University Hospital Basel (Switzerland), as previously detailed.^8^ A single day rest/stress protocol using 400/800 MBq of ^99m^Tc-sestamibi was performed.^8^ Stress was provoked by either symptom-limited bicycle exercise (*n* = 305) or adenosine vasodilator infusion (*n* = 95) while vital signs and electrocardiogram were monitored. SPECT images were visually scored by two experienced readers blinded to clinical data. A visual semi-quantitative 17-segment model with a 5-point scale (0: normal tracer uptake, 4: no tracer uptake) was used to calculate summed stress (SSS), rest (SRS) and summed difference score ([SDS] = SSS–SRS) as proposed by the American Society of Nuclear Cardiology.^14^ Abnormal SPECT was defined as SSS ≥ 4, SDS ≥ 2, or both. Normal left ventricular ejection fraction (LVEF) was defined as ≥50%.

### Endpoints

The primary endpoint was all-cause mortality. Secondary endpoints included cardiovascular mortality, MACE, and MACE + late revascularization. MACE included cardiovascular mortality and non-fatal myocardial infarction. Late revascularization was defined as clinically indicated percutaneous coronary intervention (PCI) or coronary artery bypass grafting (CABG) performed after the initially scheduled revascularization at enrolment. In cases of multiple endpoints, the first event was recorded.

### Statistical analysis

Normally distributed continuous variables are reported as mean ± standard deviation (SD), and statistical testing was performed with unpaired *t*-test or ANOVA. Non-normally distributed continuous variables are reported as median ± inter-quartile range (IQR) and Mann–Whitney *U* or Kruskal–Wallis tests were used where appropriate. Categorical variables are displayed using frequencies and percentages and were compared using the χ^2^ test or Fisher’s exact text where appropriate. Baseline characteristics were stratified according to vital status at closure of database and SPECT result. The prevalence of metabolic syndrome was calculated according to the definition of the National Cholesterol Education Program ATP III 2005.

Difference in the event-free survival was calculated using the Kaplan–Meier method, stratified by abnormal SPECT, ‘per protocol’ and ‘as treated’ status, abnormal LVEF and presence of metabolic syndrome. The log-rank test was used to test for statistical significance. Clinically relevant variables were analysed using the univariate cox regression analysis. If *P*-value was <0.05, statistically significant variables were included in a multivariate regression model. Different SPECT-derived variables were tested in univariate cox regression analysis, but not used in the multivariate model due to obvious collinearity with ‘abnormal SPECT’. Event rates were calculated by dividing the number of events by the median follow-up duration.

A *P*-value <0.05 was considered statistically significant. SPSS Version 28 and R version 4.3.1 were used for statistical analysis and plotting.

## Results

### Patient characteristics

Patient characteristics are summarized in *Table 1*. The majority of patients were male (69%) with a mean age of 63 ± 8 years. Most patients (87%) met the inclusion criteria of high coronary risk by demonstrating end-organ damage. Overweight and obesity were present in 86 and 48% of patients, respectively. The median T2DM duration was 9 (5, 15) years and patients were on oral glucose-lowering drugs and insulin in 82 and 52%, respectively. Only 44% had adequately controlled blood glucose levels at enrolment (HbA1c < 7%).^9^ Additionally, 47% reported shortness of breath. Abnormal SPECT findings, myocardial scar, and ischaemia were observed in 22, 15, and 15% of patients, respectively. Patients who died during follow-up were older, had a longer T2DM duration, smoked more frequently, and had more end-organ damage (peripheral artery disease, renal insufficiency, peripheral and cardiac autonomic neuropathy, and erectile dysfunction).

**Table 1:** Baseline characteristics

	Overall	Alive	Dead	*P*-value
*n*=	400	303	97	
Male sex (%)	275 (68.8)	207 (68.3)	68 (70.1)	0.838
Age	62.3 (7.6)	61.4 (7.6)	65.3 (6.9)	**<0.001**
Diabetes duration (years)	10.4 (7.5)	10.0 (6.9)	11.9 (9.0)	**0.029**
Dyslipidemia (%)	326 (81.5)	250 (82.5)	76 (78.4)	0.443
Hypertension (%)	346 (86.5)	263 (86.8)	83 (85.6)	0.890
Family history (%)	79 (19.8)	58 (19.1)	21 (21.6)	0.694
Smoker	230 (57.5)	160 (52.8)	70 (72.2)	**0.001**
Dyspnea (%)	187 (46.8)	135 (44.6)	52 (53.6)	0.150
Number of cardiovascular risk factors	3.5 (0.8)	3.4 (0.8)	3.6 (0.8)	0.091
**End-organ damage**				
Diabetic end-organ damage (%)	349 (87.2)	259 (85.5)	90 (92.8)	0.089
Abnormal ECG (%)	53 (13.2)	36 (11.9)	17 (17.5)	0.209
Peripheral artery disease (%)	71 (17.8)	43 (14.2)	28 (28.9)	**0.002**
Cerebral artery disease (%)	43 (10.8)	35 (11.6)	8 (8.2)	0.468
Microalbuminuria (%)	181 (45.2)	133 (43.9)	48 (49.5)	0.595
Retinopathy (%)	93 (23.2)	69 (22.8)	24 (24.7)	0.794
Peripheral neuropathy (%)	194 (48.5)	135 (44.6)	59 (60.8)	**0.003**
Cardiac autonomic neuropathy (%)	175 (43.8)	118 (38.9)	57 (58.8)	**<0.001**
Erectile dysfunction (%)	133 (33.2)	92 (30.4)	41 (42.3)	**0.033**
**Medication**				
Oral antidiabetic medication (%)	328 (82.0)	251 (82.8)	77 (79.4)	0.536
Duration of oral antidiabetic therapy (years)	8.0 (7.1)	7.6 (6.4)	9.3 (8.6)	0.050
Insulin therapy (%)	206 (51.5)	150 (49.5)	56 (57.7)	0.196
Duration insulin therapy (years)	6.1 (6.9)	5.6 (6.5)	7.4 (7.8)	0.094
Antiplatelet therapy (%)	210 (52.5)	149 (49.2)	61 (62.9)	**0.025**
Oral anticoagulation (%)	19 (4.8)	14 (4.6)	5 (5.2)	1.000
Betablocker (%)	128 (32.0)	86 (28.4)	42 (43.3)	**0.009**
Calcium antagonist (%)	101 (25.2)	72 (23.8)	29 (29.9)	0.282
Nitrate	2 (0.5)	1 (0.3)	1 (1.0)	0.980
Lipid lowering therapy (%)	229 (57.2)	172 (56.8)	57 (58.8)	0.820
Renin angiotensin aldosterone system blocking agent (%)	306 (76.5)	232 (76.6)	74 (76.3)	1.000
Diuretic (%)	195 (48.8)	136 (44.9)	59 (60.8)	**0.009**
**Physical examination**				
BMI (kg/m^2^)	30.6 (5.8)	30.6 (5.7)	30.7 (6.1)	0.840
Resting heart rate (bpm)	74.5 (11.6)	73.6 (11.2)	77.4 (12.3)	**0.005**
Resting systolic blood pressure (mmHg)	137.7 (18.4)	137.0 (18.2)	139.9 (19.0)	0.182
Resting diastolic blood pressure (mmHg)	76.1 (9.8)	76.4 (9.6)	75.1 (10.6)	0.274
**Laboratory**				
Glucose (mmol/L)	7.9 (2.6)	8.0 (2.6)	7.7 (2.6)	0.325
HbA1c (%)	7.3 (1.2)	7.3 (1.2)	7.3 (1.2)	0.862
Creatinin (umol/L)	77.2 (24.2)	73.9 (21.3)	87.7 (29.1)	**<0.001**
Estimated glomerular filtration rate (mL/min)	114.0 (40.5)	118.3 (38.7)	100.6 (43.0)	**<0.001**
Total cholesterol (mmol/L)	4.6 (1.1)	4.7 (1.1)	4.5 (1.1)	0.225
LDL (mmol/L)	2.5 (0.9)	2.5 (0.9)	2.3 (0.9)	0.140
HDL (mmol/L)	1.3 (0.4)	1.2 (0.4)	1.3 (0.4)	0.350
Triglycerides (mmol/L)	2.1 (1.5)	2.1 (1.2)	2.2 (2.1)	0.467
BNP (ng/L)	37.0[18.9,77.2]	34.6[17.3,64.8]	50.1[26.2,112.0]	**0.001**
**ECG**				
Sinus rhythm (%)	393 (98.2)	299 (98.7)	94 (96.9)	0.475
Q wave (%)	19 (4.8)	14 (4.6)	5 (5.2)	1.000
Repolarization abnormality (%)	88 (22.0)	60 (19.8)	28 (28.9)	**0.083**
**SPECT**				
Antianginal medication stopped (%)	373 (93.2)	281 (92.7)	92 (94.8)	0.626
Physical stress (%)	305 (76.2)	241 (79.5)	64 (66.0)	**0.010**
Scar on SPECT	58 (14.5)	37 (12.2)	21 (21.6)	**0.033**
Ischaemia	61 (15.2)	40 (13.2)	21 (21.6)	0.064
SRS	0.0[0.0,1.0]	0.0[0.0,0.0]	0.0[0.0,2.0]	**0.004**
SDS	0.0[0.0,0.0]	0.0[0.0,0.0]	0.0[0.0,0.0]	0.087
SSS	0.0[0.0,2.0]	0.0[0.0,1.0]	0.0[0.0,6.0]	**0.004**
Rest LVEF (%)	57.8 (10.6)	58.1 (10.0)	56.7 (12.4)	0.269

Baseline characteristics stratified by vital status. Values displayed as mean (SD), median (IQR), or number (%) where appropriate. Significant *P*-values are marked in bold.

### Randomization

Eighty-seven (22%) patients with an abnormal baseline SPECT were randomized to either conservative (*n* = 41) or invasive treatment (*n* = 46). In the invasive strategy arm, 5 patients refused invasive angiography, and 11 were not suitable for revascularization. Thus, 30 patients (65%) successfully underwent index revascularization as scheduled at baseline (21 PCI, 9 CABG).

### Ten-year follow-up

The median follow-up was 11.1 (8.8, 12.8) years. During this time, 97 patients (24%) died, with a median time to death of 8.6 (5.5, 11.6) years. Cardiovascular mortality accounted for 34 patients (35% of all deaths, 9% overall). A non-fatal myocardial infarction was observed in 42 patients (11%) after median 5.5 (3.1, 8.6) years. Patients were revascularized with PCI or CABG in 13 and 6%, respectively. During the study, 35 patients (9%) were lost to follow-up. Three hundred and eighteen patients (79.5%) completed at least 10 years of follow-up or died before. Event rates, stratified by baseline SPECT results, are presented in *Table 2*.

**Table 2:** Endpoints during 10 years follow-up

	Normal SPECT		Abnormal SPECT		*P*-value
	*n* = 313	Events/year (%)	*n* = 87	Events/year (%)	
All-cause mortality	67 (21%)	1.9	30 (35%)	3.1	0.016
Cardiovascular mortality	19 (6%)	0.6	15 (17%)	1.5	0.002
MACE	39 (13%)	1.2	21 (24%)	2.3	0.010
MACE + revascularization	57 (18%)	1.7	31 (36%)	3.7	0.001

Occurrence and events-rates of endpoints stratified by baseline SPECT result. Number of patients with percentage in brackets. Fisher’s exact test was used to compare between groups.

### Event rates depending on baseline SPECT

**Figure 1:**
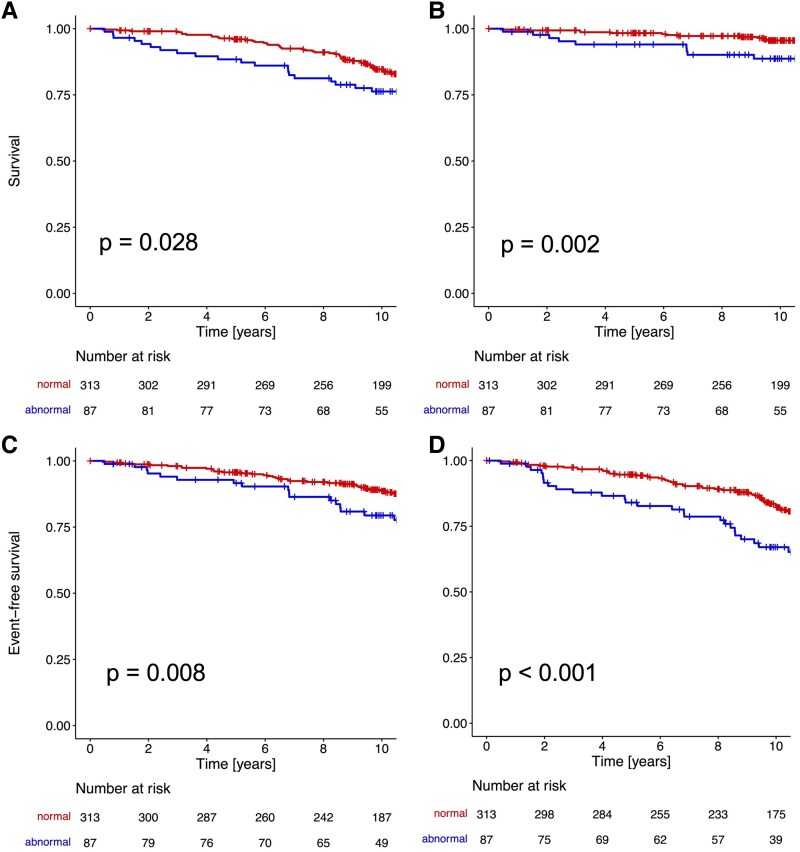
Kaplan–Meier curve analysis stratified by SPECT result. Kaplan–Meier survival curves stratified by normal (SSS < 4 and SDS < 2) vs. abnormal (SSS ≥ 4 or SDS ≥ 2) SPECT for the following endpoints: all-cause mortality (*A*), cardiovascular mortality (*B*), MACE (*C*), and MACE + revascularization (*D*).

As shown in *Figure 1*, event-free survival was statistically better for all endpoints in patients with normal baseline SPECT compared to those with abnormal SPECT. The greatest difference was observed in the MACE + revascularization endpoint.

### Predictors of all-cause mortality

Predictors for all-cause mortality are summarized in *Table 3*. In univariate analysis, abnormal SPECT, age, smoking status, peripheral artery disease, peripheral neuropathy, cardiac autonomic neuropathy, erectile dysfunction, diastolic blood pressure, creatinine, and B-type natriuretic peptide (BNP) levels, and the inability to perform physical stress test were associated with increased all-cause mortality. Additional significant predictors derived from SPECT were SSS, SRS, and the presence of scar. In the multivariate model, adjusting for all significant variables, only age, cardiac autonomic neuropathy, BNP levels, and the ability to perform the physical stress test remained significant. Abnormal SPECT was no longer a significant predictor.

**Table 3:** Cox regression analysis of different variables for all-cause mortality

All-cause mortality	Univariate				Multivariate			
	HR	95% CI		*P*-value	HR	95% CI		*P*-value
Abnormal SPECT (SSS ≥ 4 or SDS ≥ 2)	1.614	1.049	2.482	**0.029**	0.915	0.549	1.526	0.734
Male sex	1.009	0.653	1.559	0.967				
Age	1.079	1.045	1.114	**<0.001**	1.068	1.027	1.110	**0.001**
Diabetes duration	1.024	0.999	1.050	0.060				
Diabetes end-organ damage	2.000	0.927	4.317	0.077				
Duration insulin	1.029	0.994	1.066	0.104				
Oral antidiabetic drug duration	1.019	0.992	1.048	0.173				
Hypertension	0.894	0.507	1.575	0.698				
Family history	1.169	0.721	1.895	0.527				
Smoker	1.986	1.273	3.096	**0.002**	1.619	0.890	2.945	0.115
Number of cardiovascular risk factors	1.191	0.930	1.525	0.166				
Peripheral artery disease	1.822	1.174	2.829	**0.007**	1.220	0.713	2.087	0.468
Cerebral artery disease	0.730	0.354	1.506	0.395				
Microalbuminuria	1.162	0.777	1.738	0.466				
Retinopathy	1.292	0.814	2.051	0.277				
Peripheral neuropathy	1.717	1.138	2.592	**0.010**	1.124	0.684	1.848	0.644
Cardiac autonomic neuropathy	2.282	1.502	3.468	**<0.001**	2.187	1.305	3.667	**0.003**
Erectile dysfunction	1.659	1.056	2.608	**0.028**	1.424	0.859	2.362	0.171
Dyspnoea	1.402	0.941	2.090	0.097				
BMI (kg/m^2^)	1.009	0.975	1.044	0.604				
Systolic blood pressure (mmHg)	0.999	0.989	1.010	0.915				
Diastolic blood pressure (mmHg)	0.968	0.947	0.989	**0.003**	0.986	0.959	1.014	0.315
HbA1c (%)	0.966	0.817	1.142	0.686				
Creatinine (µmol/L)	1.015	1.009	1.022	**<0.001**	1.005	0.996	1.013	0.293
Total cholesterol (mmol/L)	0.843	0.696	1.020	0.079				
LDL (mmol/L)	0.825	0.656	1.037	0.099				
HDL (mmol/L)	1.088	0.666	1.776	0.736				
BNP (pg/mL)	1.003	1.002	1.003	**<0.001**	1.002	1.001	1.003	**0.005**
Q wave	1.303	0.529	3.209	0.565				
Repolarization abnormality	1.267	0.815	1.969	0.293				
Physical exercise possible	0.356	0.237	0.536	**<0.001**	0.459	0.271	0.777	**0.004**
Symptoms on exercise	1.284	0.741	2.227	0.373				
Scar	1.669	1.029	2.706	**0.038**				
Ischaemia	1.542	0.951	2.501	0.079				
SSS	1.045	1.006	1.086	**0.025**				
SDS	1.026	0.951	1.107	0.501				
SRS	1.083	1.024	1.144	**0.005**				
Resting LVEF (%)	0.987	0.968	1.006	0.187				
Post-stress LVEF (%)	0.986	0.967	1.005	0.151				

Results of univariate Cox regression analysis for all-cause mortality are displayed on the left side. Significant variables with *P* < 0.05 were used in the multivariate model displayed on the right. Significant *P*-values are marked in bold.

### Predictors of cardiovascular mortality

Univariate analysis identified abnormal SPECT, age, peripheral artery disease, cardiac autonomic neuropathy, creatinine, and BNP levels, repolarization abnormalities, post-stress LVEF and the ability to perform physical stress tests as significant predictors of cardiovascular mortality. SPECT-related predictors included SSS, SRS, SDS, and the presence of ischaemia. In the multivariate model, only age, peripheral artery disease, cardiac autonomic neuropathy, creatinine, and BNP levels, and the ability to perform physical stress test remained significant. Details are displayed in Supplementary data online, *Table S1*.

### Predictors of MACE

In univariate analysis, significant predictors of MACE included abnormal SPECT, age, peripheral artery disease, peripheral neuropathy, cardiac autonomic neuropathy, creatinine, and BNP levels, and the ability to perform physical stress test. Additional significant variables derived from SPECT were SSS, SDS the presence of ischaemia. In the multivariate model, only age, cardiac autonomic neuropathy, creatinine levels, and the ability to perform physical stress test remained significant predictors. Details are displayed in Supplementary data online, *Table S2*.

### Predictors of MACE ± late revascularization

In univariate analysis, significant predictors for MACE + late revascularization included abnormal SPECT, age, number of cardiovascular risk factors, peripheral artery disease, cardiac autonomic neuropathy, diastolic blood pressure, creatinine, and BNP levels, repolarization abnormalities, and the ability to perform physical stress test. SPECT-related predictors included SSS, SRS, SDS, and the presence of ischaemia. In the multivariate model, only age, number of cardiovascular risk factors, and the ability to perform physical stress test remained significant predictors. Details are displayed in Supplementary data online, *Table S3*.

### Event-free survival depending on revascularization strategy (‘per protocol’)

Event-free survival was highest in patients with normal SPECT, compared to those with abnormal SPECT who were randomized to either revascularization or conservative treatment. The overall trend was statistically significant for cardiovascular mortality (*P* = 0.007), MACE (*P* = 0.029), and MACE + late revascularization (*P* < 0.001), but not for all-cause mortality. Pairwise comparisons are depicted in *Table 4*. There was no significant difference between patients randomized to the invasive vs. conservative treatment strategy.

**Table 4:** Pairwise comparison of Kaplan–Meier curves depending on SPECT result and randomization status (‘per protocol’)

	All-cause mortality	Cardiovascular mortality	MACE	MACE + revascularization
Normal vs. Invasive	0.086	**0.012**	**0.047**	0.066
Normal vs. Conservative	0.087	**0.010**	**0.029**	**<0.001**
Invasive vs. Conservative	0.953	1.000	0.888	0.091

The *P*-values (log-rank test) from pairwise comparisons of Kaplan–Meier curves are displayed for all clinical endpoints. Significant *P*-values are marked in bold.

### Event-free survival depending on revascularization strategy (‘as treated’)

**Figure 2:**
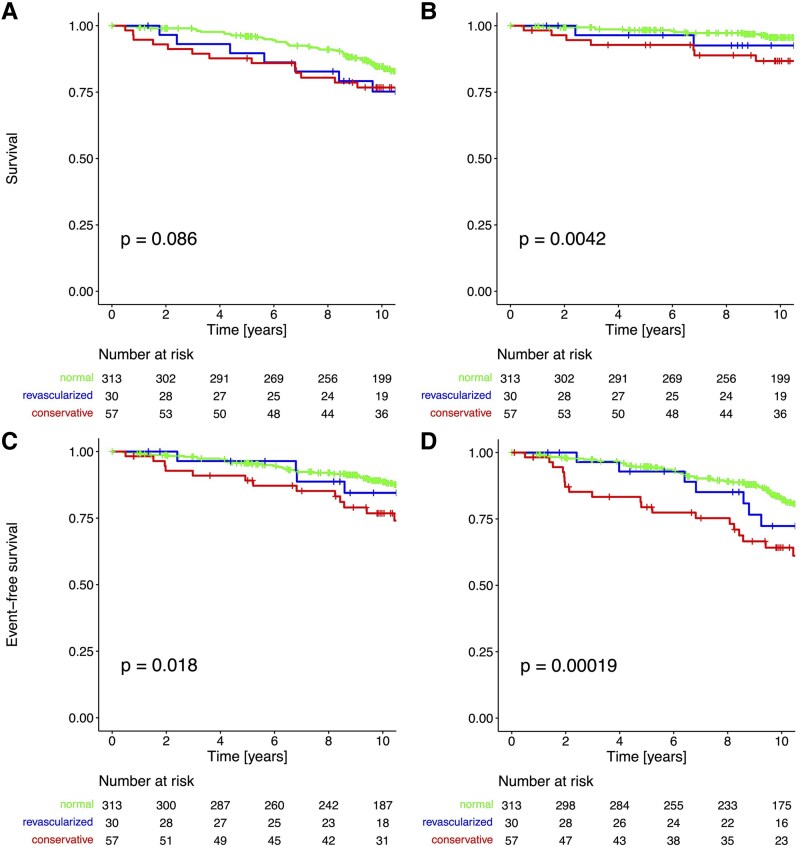
Kaplan–Meier curve analysis depending on SPECT result and index treatment. Kaplan–Meier survival curves stratified by SPECT result and performed index strategy (revascularized vs. conservative or revascularization not possible). The four panels represent the following endpoints: all-cause mortality (*A*), cardiovascular mortality (*B*), MACE (*C*), and MACE + revascularization (*D*).

As shown in *Figure 2*, event-free survival was best in patients with normal SPECT, followed by those who underwent revascularization. Patients treated conservatively had the worst event-free survival. The *P*-value for the overall trend was significant for all endpoints except for all-cause mortality. As shown in *Table 5*, the only significant difference was between patients with normal SPECT and those with abnormal SPECT treated conservatively. However, among patients with abnormal SPECT, there was no statistically significant difference in event-free survival between revascularization and conservative treatment.

**Table 5:** Pairwise comparison of Kaplan–Meier curves depending on SPECT result and index revascularization status (‘as treated’)

	All-cause mortality	Cardiovascular mortality	MACE	MACE + revascularization
Normal vs. abnormal (revascularized)	0.216	0.180	0.298	0.143
Normal vs. abnormal (conservative)	**0.042**	**<0.001**	**0.005**	**<0.001**
Abnormal (revascularized) vs. abnormal (conservative)	0.827	0.470	0.430	0.204

The *P*-value from pairwise comparison of Kaplan–Meier curves is displayed for all clinical endpoints. Significant *P*-values are marked in bold.

### Event-free survival depending on LVEF and metabolic syndrome

No rest LVEF cut-off (LVEF ≥ 50% or tertiles) was predictive for any endpoint (see Supplementary data online, *Table S5*). However, abnormal post-stress LVEF (<50%) was a significant predictor for overall mortality [hazard ratio (HR) 1.7, *P* = 0.023], cardiovascular mortality (HR 2.8, *P* = 004), and MACE + revascularization (HR 1.9, 0.007). There was a non-significant trend for better outcome in patients with post-stress LVEF 56–63% compared with <56 and ≥64%.

Metabolic syndrome was present in 91% of patients. The majority of patients fulfilled 4 or more out of the 5 diagnostic criteria (4 in 38%, all in 31%). The presence or absence of metabolic syndrome was not predictive for any endpoint.

### Factors associated with an abnormal SPECT result

As demonstrated in Supplementary data online, *Table S4*, several baseline characteristics were significantly associated with abnormal SPECT. Patients with abnormal SPECT were more likely to be male, older, had a longer duration of diabetes, and be smokers. They also had a higher number of cardiovascular risk factors, peripheral artery disease, peripheral neuropathy, cardiac autonomic neuropathy, erectile dysfunction, duration of T2DM treatment, antiplatelet therapy, creatinine and BNP levels, and reduced physical capacity. Most of these factors were also associated with poorer prognosis, as described above.

## Discussion

The primary finding of this study in high-risk T2DM patients is that a normal SPECT scan was associated with a good long-term prognosis over 10 years. A normal scan result was associated with persistently lower rates of MACE (1.2%/year) and a very low rate of cardiovascular mortality (0.6%/year). Despite the fact that 87% of our cohort had diabetes with end-organ damage, a normal SPECT scan provided a reassuring prognosis over 10 years, similar to that of the normal population. These findings align with a large meta-analysis of 13 493 SPECT scans in diabetic patients, where the annual MACE rate of a normal scan was also 1.2%/year.^15^ Our study adds to this by providing long-term data, as the mean follow-up in the meta-analysis was only 3 years.

In the univariate analysis, several SPECT-derived variables were predictive of all-cause mortality, cardiovascular mortality, MACE, and MACE + late revascularization. However, after adjusting for other significant ‘non-SPECT’ variables, abnormal SPECT and SPECT-derived variables were no longer independent predictors of any endpoint. This suggests a close association between SPECT results and other factors that predicted poor outcome such as age, male sex, smoking, T2DM duration, diabetic end-organ damage (autonomic neuropathy, peripheral artery disease), creatinine, and BNP levels.

This might appear disappointing at first sight; but the prognostic value of SPECT must not be narrowed over hastily. Although the continuous variables (creatinine, BNP, age) carried more weight in the multivariate model, they are influenced by both age and gender. Consequently, specific cut-off values tailored to this cohort would be necessary for their effective use, which would require further internal and external validation in high-risk T2DM patients. Because many well-known risk factors and prognostically relevant biomarkers were associated with an abnormal SPECT (detailed in Supplementary data online, *Table S4*), a SPECT scan could be regarded as holistic integration of them and be used as an easy-to-interpret surrogate marker of cardiovascular risk providing a simple binary response (patient at risk or not).

Of note, patients who were able to perform the physical stress test, had less often an abnormal SPECT and a 54% lower risk of all-cause mortality over 10 years. This highlights the importance of physical fitness also in diabetic patients.

An abnormal post-stress LVEF < 50% was predictive for all endpoints except for MACE. This might be a surrogate of higher ischaemia burden as described for transient dilatation which is also associated with worse prognosis.^16^ Rest LVEF was not predictive for any endpoint.

Although screening asymptomatic T2DM patients has not been shown to reduce cardiovascular events in previous randomized controlled trials,^17–21^ cardiac imaging still incorporates important prognostic information: in the DIAD trial, patients with no or small perfusion defects on SPECT had significantly lower event rates compared with moderate or larger defects (0.4%/year vs. 2.4%/year, *P* = 0.001).^17^ Consistent with our study, unadjusted event-free survival was better in patients with normal SPECT scans. Therefore, our findings align with previous studies highlighting the prognostic value of SPECT in T2DM patients.

Two large randomized imaging trials showed no statistically significant reduction of MACE by screening (DIAD^17^ HR 0.88, *P* = 0.73; FACTOR-64^19^ HR 0.8, *P* = 0.38). One possible reason for the non-significant benefit of advanced screening could be the lower-than-expected event rates or the limited follow-up duration in the cohorts (DIAD 4.8 years, FACTORS-64 4.0 years). A meta-analysis of five randomized controlled trials comprising 3299 asymptomatic diabetic patients showed a 27% relative reduction in cardiac events (HR 0.73, *P* = 0.028) after a mean follow-up of 4.1 years.^11^ This reduction was primarily driven by trends towards fewer non-fatal myocardial infarctions (HR 0.65, *P* = 0.062) and heart failure hospitalizations (HR 0.61, *P* = 0.100), but not cardiac mortality (HR 0.92, *P* = 0.77). It remains speculative whether a longer follow-up or larger sample size would have resulted in different outcomes in the cited trials.

For example, after a median 9.8 years of follow-up in the extended STICH trial, CABG was shown to be superior compared with medical treatment alone. This was also observed in the subgroup of diabetic patients, but this trend did not reach statistical significance. Hence, screening of high-risk diabetic patients should not be abandoned in clinical routine.

As shown in this study and previous publications from BARDOT publications, SPECT remains a robust tool for risk stratification in asymptomatic high-risk T2DM patients.^6–8^ Current guidelines do not recommend routine CAD screening in diabetic patients due to a lack of proven prognostic benefit.^9^ However, these recommendations are based on trials using different non-invasive modalities with a limited follow-up duration. With a median follow-up duration of over 11 years, this study expands current knowledge affirming the long-term prognostic value of SPECT, especially when the results are normal.

### Limitations

The study was initiated before coronary artery calcium score (CACS) was routinely used, so we were unable to assess the complementary diagnostic and prognostic value of CACS. Moreover, advances in image reconstruction techniques, such as attenuation and scatter correction, were not available during recruitment. However, it is unlikely that newer protocols would have significantly altered the results, as prone positioning was used to enhance specificity and all images were reviewed by highly experienced readers (>10 years). As attenuation correction was not performed at the time of recruitment, no CT images for retrospective CACS calculation were available.

Coronary microvascular dysfunction and reduced myocardial flow reserve are very common in asymptomatic diabetic patients and associated with adverse outcome.^4^ Unfortunately, this valuable tool was not available when this study was planned and performed. If the study was to be repeated, Positron Emission Tomography would be used nowadays with which myocardial blood flow is routinely assessed. This would allow advanced risk stratification and prognostication.

Another limitation is the high prevalence of non-cardiac death in this cohort of high-risk diabetics, which creates a competing risk. A non-diabetic control group would have been helpful to differentiate the risks, but ethically, it would not have been justified to perform SPECT on asymptomatic non-diabetic patients.

Finally, the relatively small number of patients randomized, and the low event rate may limit statistical power to assess the effect of revascularization in this small subgroup.

## Conclusion

A normal SPECT is associated with a favourable long-term prognosis in asymptomatic high-risk T2DM patients. Since several prognostically important factors are associated with an abnormal SPECT finding, a SPECT scan could be considered as an optimal surrogate marker for advanced risk stratification. Importantly, a normal scan result can identify patients at low risk who were initially thought to be at highest risk due to T2DM with organ damage, making it a valuable tool for risk assessment.

## Supplementary Material

jeaf126_Supplementary_Data

## Data Availability

Data available upon reasonable request.
