# Supplementary material for: Prognostic value of myocardial perfusion imaging in asymptomatic high-risk diabetic patients: 10-year follow-up of the prospective multicentre BARDOT trial
Source: Eur Heart J Cardiovasc Imaging. 2025 Apr 23;26(7):1130–9. doi: 10.1093/ehjci/jeaf126 (PMC12206573; doi:10.1093/ehjci/jeaf126)
Supplement: jeaf126_Supplementary_Data [file jeaf126_supplementary_data.pdf]

Appendix

Supplemental Table S1: Cox regression analysis of different variables for cardiovascular death

Supplemental Table S2: Cox regression analysis of different variables for MACE

Supplemental Table S3: Cox regression analysis of different variables for MACE and revascularization

Supplemental Table 4: Difference in baseline characteristics according to SPECT result

Supplemental Table 5: Predictive value of LVEF for endpoints

Supplemental Table S1: Cox regression analysis of different variables for cardiovascular death

**Cardiovascular death**	**Univariate**				**Multivariate**			
	HR	95% CI		p-value	HR	95% CI		p-value
Abnormal SPECT (SSS ≥4 or SDS ≥2)	2.820	1.433	5.553	**0.003**	1.257	0.559	2.827	0.580
Male sex	0.862	0.402	1.848	0.703				
Age	1.102	1.042	1.166	**0.001**	1.083	1.020	1.148	**0.008**
Diabetes duration	1.018	0.975	1.062	0.418				
Diabetes end organ damage	2.426	0.581	10.127	0.224				
Duration insulin	1.014	0.951	1.082	0.663				
OAD duration	1.023	0.975	1.072	0.352				
Hypertension	0.879	0.340	2.273	0.791				
Family history	1.511	0.705	3.237	0.289				
Smoker	1.374	0.680	2.778	0.376				
Number of cardiovascular risk factors	1.315	0.860	2.011	0.206				
Peripheral artery disease	5.017	2.556	9.848	**<0.001**	2.379	1.130	5.008	**0.022**
Cerebral artery disease	1.441	0.557	3.731	0.451				
Microalbuminuria	1.388	0.704	2.737	0.343				
Retinopathy	1.435	0.668	3.082	0.354				
Peripheral neuropathy	2.093	1.014	4.320	0.046				
Cardiac autonomic neuropathy	3.849	1.774	8.349	**<0.001**	2.909	1.278	6.621	**0.011**
Erectile dysfunction	1.946	0.919	4.121	0.082				
Dyspnea	1.959	0.981	3.915	0.053				
BMI (kg/m2)	1.048	0.995	1.105	0.078				
syst. BD (mmHg)	1.008	0.990	1.026	0.378				
diast. BD (mmHg)	0.972	0.938	1.009	0.135				
HbA1c (%)	1.053	0.813	1.364	0.696				
Kreatinin (umol/l)	1.020	1.011	1.030	**<0.001**	1.012	1.000	1.024	**0.046**
Total cholesterol (mmol/l)	1.002	0.734	1.368	0.991				
LDL (mmol/l)	0.945	0.647	1.379	0.767				
HDL (mmol/l)	0.900	0.383	2.113	0.809				
BNP (pg/ml)	1.003	1.002	1.004	**<0.001**	1.002	1.000	1.003	**0.027**
Q wave	1.491	0.356	6.246	0.585				
Repolarisation abnormality	2.099	1.055	4.174	**0.035**	1.203	0.557	2.599	0.638
Physical exercise possible	0.113	0.047	0.275	**<0.001**	0.225	0.087	0.580	**0.002**
Symptoms on exercise	1.214	0.470	3.137	0.689				
Scar	1.550	0.674	3.561	0.302				
Ischemia	3.402	1.703	6.796	**0.001**				
SSS	1.085	1.031	1.143	**0.002**				
SDS	1.108	1.028	1.193	**0.007**				
SRS	1.109	1.018	1.208	**0.017**				
Resting LVEF (%)	0.976	0.945	1.008	0.134				
Post-stress LVEF (%)	0.968	0.938	0.999	0.048	0.994	0.960	1.030	0.753

Results of univariate Cox regression analysis for cardiovascular death are displayed on the left side. Significant variables with $p < 0.05$ were used in the multivariate model displayed on the right.

Supplemental Table S2: Cox regression analysis of different variables for MACE

**MACE**	**Univariate**				**Multivariate**			
	HR	95% CI		p-value	HR	95% CI		p-value
Abnormal SPECT (SSS ≥4 or SDS ≥2)	2.024	1.190	3.441	**0.009**	1.235	0.698	2.185	0.468
Male sex	1.108	0.643	1.911	0.711				
Age	1.076	1.034	1.120	**0.001**	1.062	1.020	1.105	**0.003**
Diabetes duration	1.024	0.992	1.057	0.140				
Diabetes end organ damage	1.686	0.675	4.214	0.264				
Duration insulin	1.026	0.979	1.075	0.283				
OAD duration	1.012	0.976	1.050	0.513				
Hypertension	1.760	0.704	4.397	0.226				
Family history	1.033	0.548	1.944	0.921				
Smoker	1.149	0.685	1.928	0.598				
Number of cardiovascular risk factors	1.203	0.883	1.640	0.242				
Peripheral artery disease	2.350	1.373	4.022	**0.002**	1.497	0.830	2.699	0.180
Cerebral artery disease	1.660	0.841	3.277	0.144				
Microalbuminuria	1.351	0.813	2.246	0.245				
Retinopathy	0.920	0.488	1.733	0.796				
Peripheral neuropathy	2.154	1.256	3.695	**0.005**	1.363	0.779	2.387	0.278
Cardiac autonomic neuropathy	2.484	1.451	4.252	**<0.001**	2.057	1.180	3.587	**0.011**
Erectile dysfunction	1.581	0.891	2.806	0.117				
Dyspnea	1.172	0.707	1.945	0.538				
BMI (kg/m2)	1.000	0.957	1.044	0.998				
syst. BD (mmHg)	1.005	0.992	1.019	0.434				
diast. BD (mmHg)	0.973	0.948	1.000	0.050				
HbA1c (%)	1.010	0.823	1.240	0.924				
Kreatinin (umol/l)	1.017	1.009	1.025	**0.001**	1.011	1.002	1.020	**0.020**
Total cholesterol (mmol/l)	1.144	0.909	1.439	0.252				
LDL (mmol/l)	1.203	0.916	1.580	0.185				
HDL (mmol/l)	0.963	0.508	1.826	0.907				
BNP (pg/ml)	1.002	1.001	1.003	**0.001**	1.001	0.999	1.002	0.432
Q wave	1.190	0.372	3.804	0.770				
Repolarisation abnormality	1.539	0.892	2.656	0.121				
Physical exercise possible	0.329	0.194	0.558	**<0.001**	0.489	0.278	0.863	**0.013**
Symptoms on exercise	1.626	0.845	3.129	0.145				
Scar	1.036	0.510	2.105	0.922				
Ischemia	2.566	1.477	4.459	**0.001**				
SSS	1.065	1.020	1.113	**0.005**				
SDS	1.102	1.035	1.174	**0.003**				
SRS	1.068	0.993	1.149	0.078				
Resting LVEF (%)	1.003	0.979	1.028	0.798				
Post-stress LVEF (%)	0.994	0.970	1.018	0.607				

Results of univariate Cox regression analysis for MACE are displayed on the left side. Significant variables with $p < 0.05$ were used in the multivariate model displayed on the right.

Supplemental Table S3: Cox regression analysis of different variables for MACE and revascularization

**MACE + late revascularization**	**Univariate**				**Multivariate**			
	HR	95% CI		p-value	HR	95% CI		p-value
Abnormal SPECT (SSS ≥4 or SDS ≥2)	2.289	1.478	3.546	**<0.001**	1.442	0.893	2.329	0.135
Male sex	0.840	0.526	1.344	0.468				
Age	1.060	1.027	1.094	**<0.001**	1.053	1.019	1.089	**0.002**
Diabetes duration	1.008	0.981	1.036	0.560				
Diabetes end organ damage	1.338	0.671	2.668	0.408				
Duration insulin	1.017	0.976	1.060	0.422				
OAD duration	1.004	0.974	1.036	0.782				
Hypertension	2.237	0.976	5.124	0.057				
Family history	0.904	0.526	1.555	0.716				
Smoker	1.432	0.927	2.212	0.105				
Number of cardiovascular risk factors	1.364	1.054	1.766	**0.018**	1.435	1.076	1.915	**0.014**
Peripheral artery disease	2.205	1.402	3.470	**<0.001**	1.371	0.836	2.246	0.211
Cerebral artery disease	1.439	0.798	2.594	0.226				
Microalbuminuria	1.335	0.878	2.030	0.176				
Retinopathy	0.727	0.417	1.268	0.261				
Peripheral neuropathy	1.196	0.785	1.823	0.404				
Cardiac autonomic neuropathy	1.543	1.006	2.366	**0.047**	1.331	0.852	2.080	0.209
Erectile dysfunction	1.375	0.866	2.184	0.177				
Dyspnea	1.042	0.686	1.584	0.847				
BMI (kg/m2)	0.986	0.950	1.023	0.439				
syst. BD (mmHg)	1.007	0.996	1.018	0.203				
diast. BD (mmHg)	0.977	0.956	0.999	**0.044**	0.984	0.961	1.008	0.199
HbA1c (%)	0.950	0.796	1.135	0.574				
Kreatinin (umol/l)	1.012	1.004	1.019	**0.002**	1.005	0.996	1.013	0.268
Total cholesterol (mmol/l)	1.157	0.958	1.397	0.131				
LDL (mmol/l)	1.193	0.952	1.495	0.126				
HDL (mmol/l)	0.817	0.473	1.413	0.471				
BNP (pg/ml)	1.002	1.001	1.003	**0.001**	1.001	1.000	1.002	0.251
Q wave	1.351	0.547	3.334	0.514				
Repolarisation abnormality	1.602	1.022	2.510	**0.040**	1.341	0.817	2.200	0.245
Physical exercise possible	0.398	0.260	0.609	**<0.001**	0.571	0.359	0.909	**0.018**
Symptoms on exercise	1.461	0.838	2.547	0.182				
Scar	1.433	0.834	2.465	0.193				
Ischemia	2.280	1.416	3.669	**0.001**				
SSS	1.082	1.043	1.122	**<0.001**				
SDS	1.212	1.135	1.295	**<0.001**				
SRS	1.078	1.015	1.144	**0.014**				
Resting LVEF (%)	0.993	0.973	1.014	0.513				
Post-stress LVEF (%)	0.988	0.968	1.009	0.271				

Results of univariate Cox regression analysis for MACE and revascularization are displayed on the left side. Significant variables with $p < 0.05$ were used in the multivariate model displayed on the right.

Supplemental Table 4: Difference in baseline characteristics according to SPECT result

	**Overall**	**Normal SPECT**	**Abnormal SPECT**	**p-value**
n =	400	313	87	
Male sex (%)	275 (68.8)	202 (64.5)	73 (83.9)	**0.001**
Age	62.3 (7.6)	61.9 (7.6)	64.1 (7.2)	**0.015**
Diabetes duration [y]	10.4 (7.5)	9.8 (7.0)	12.8 (8.6)	**0.001**
Dyslipidemia (%)	326 (81.5)	255 (81.5)	71 (81.6)	1.000
Hypertension (%)	346 (86.5)	266 (85.0)	80 (92.0)	0.132
Family history (%)	79 (19.8)	67 (21.4)	12 (13.8)	0.154
Smoker	230 (57.5)	165 (52.7)	65 (74.7)	**<0.001**
Dyspnea (%)	187 (46.8)	141 (45.0)	46 (52.9)	0.241
Number of cardiovascular risk factors	3.5 (0.8)	3.4 (0.8)	3.6 (0.8)	**0.034**
**End organ damage**				
Diabetic end organ damage (%)	349 (87.2)	269 (85.9)	80 (92.0)	0.192
Abnormal ECG (%)	53 (13.2)	36 (11.5)	17 (19.5)	0.075
Peripheral artery disease (%)	71 (17.8)	42 (13.4)	29 (33.3)	**<0.001**
Cerebral artery disease (%)	43 (10.8)	31 (9.9)	12 (13.8)	0.401
Microalbuminuria (%)	181 (45.2)	135 (43.1)	46 (52.9)	0.271
Retinopathy (%)	93 (23.2)	68 (21.7)	25 (28.7)	0.220
Peripheral neuropathy (%)	194 (48.5)	144 (46.0)	50 (57.5)	**0.023**
Cardiac autonomic neuropathy (%)	175 (43.8)	125 (39.9)	50 (57.5)	**0.006**
Erectile dysfunction (%)	133 (33.2)	87 (27.8)	46 (52.9)	**<0.001**
**Medication**				
Oral antidiabetic medication (%)	328 (82.0)	253 (80.8)	75 (86.2)	0.319
Duration of oral antidiabetic therapy [y]	8.0 (7.1)	7.6 (6.5)	9.5 (8.8)	**0.035**
Insulin therapy (%)	206 (51.5)	161 (51.4)	45 (51.7)	0.530
Duration insulin therapy [y]	6.1 (6.9)	5.0 (5.6)	10.1 (9.3)	**<0.001**
Antiplatelet therapy (%)	210 (52.5)	155 (49.5)	55 (63.2)	**0.032**
Oral anticoagulation (%)	19 (4.8)	14 (4.5)	5 (5.7)	0.834
Betablocker (%)	128 (32.0)	97 (31.0)	31 (35.6)	0.489
Calcium Antagonist (%)	101 (25.2)	72 (23.0)	29 (33.3)	0.068
Nitrate	2 (0.5)	2 (0.6)	0 (0.0)	1.000
Lipid lowering therapy (%)	229 (57.2)	172 (55.0)	57 (65.5)	0.101
RAAS blocking agent (%)	306 (76.5)	233 (74.4)	73 (83.9)	0.089
Diuretic (%)	195 (48.8)	144 (46.0)	51 (58.6)	0.050
**Physical examination**				
BMI	30.6 (5.8)	30.6 (5.8)	30.7 (5.8)	0.882
Resting heart rate [bpm]	74.5 (11.6)	73.9 (11.1)	76.7 (13.0)	0.050
Resting systolic blood pressure [mmHg]	137.7 (18.4)	136.8 (18.6)	141.2 (17.5)	0.050
Resting diastolic blood pressure [mmHg]	76.1 (9.8)	76.3 (9.8)	75.3 (10.0)	0.405
**Laboratory**				
Glucose [mmol/l]	7.9 (2.6)	8.1 (2.7)	7.3 (2.4)	**0.024**
HbA1c [%]	7.3 (1.2)	7.3 (1.2)	7.4 (1.3)	0.419
Creatinin [umol/l]	77.2 (24.2)	75.1 (23.4)	84.9 (25.3)	**0.001**
GFR [ml/min]	114.0 (40.5)	115.7 (40.4)	107.7 (40.4)	0.104
Total cholesterol [mmol/l]	4.6 (1.1)	4.7 (1.0)	4.5 (1.1)	0.272
LDL [mmol/l]	2.5 (0.9)	2.5 (0.9)	2.4 (1.0)	0.330
HDL [mmol/l]	1.3 (0.4)	1.3 (0.4)	1.2 (0.4)	0.440
Triglycerides [mmol/l]	2.1 (1.5)	2.1 (1.4)	2.2 (1.8)	0.611
BNP	37.0[18.9,77.2]	33.6[18.2,66.1]	49.1[22.6,93.4]	**0.018**
**ECG**				
Sinus rhythmus (%)	393 (98.2)	308 (98.4)	85 (97.7)	1.000
Q wave (%)	19 (4.8)	15 (4.8)	4 (4.6)	1.000
Repolarisation abnormality (%)	88 (22.0)	62 (19.8)	26 (29.9)	0.063
**Stress test**				
Antianginal medication stopped (%)	373 (93.2)	293 (93.6)	80 (92.0)	0.762
Physical stress (%)	305 (76.2)	255 (81.5)	50 (57.5)	**<0.001**
Symptoms during stress test (%)	71 (17.8)	52 (16.6)	19 (21.8)	0.196
Physical capacity [W]*	122.0 (50.9)	124.7 (51.2)	111.3 (48.7)	**0.042**

Baseline characteristics stratified by SPECT result. Values displayed as mean (SD), median [IQR] or number (%) where appropriate. *in patients who were able to perform physical stress test.

Supplemental Table 5: Predictive value of LVEF for endpoints

	**Overall mortality**		**Cardiovascular mortality**		**MACE**		**MACE + revascularization**	
	HR	*p-value*	*HR*	*p-value*	*HR*	*p-value*	*HR*	*p-value*
**Rest LVEF tertiles**		0.965		0.782		0.693		0.955
≥63%	ref	ref	ref	ref	ref	ref	ref	ref
55-62%	1.008	0.974	1.017	0.969	0.773	0.426	0.932	0.794
<55%	1.062	0.807	1.287	0.543	0.821	0.524	1.001	0.996
**Rest LVEF**								
abnormal (<50%)	1.332	0.212	1.722	0.139	0.978	0.943	0.994	0.98
**Stress LVEF tertiles**		0.087		0.279		0.106		0.25
≥64%	ref	ref	ref	ref	ref	ref	ref	ref
56-63%	0.664	0.139	0.624	0.33	**0.49**	**0.038**	0.658	0.134
<56%	1.185	0.475	1.282	0.532	0.828	0.523	0.983	0.944
**Stress LVEF**								
abnormal (<50%)	**1.709**	**0.023**	**2.782**	**0.004**	1.679	0.082	**1.935**	**0.007**

Log-Rank test for different rest and post-stress LVEF cut-offs. Rest LVEF was not a significant predictor for any endpoints.
HR: hazard ratio. LVEF: left ventricular ejection fraction.
